# Fears Related to COVID-19 among Rural Older People in Japan

**DOI:** 10.3390/healthcare9050524

**Published:** 2021-04-29

**Authors:** Ryuichi Ohta, Yoshinori Ryu, Chiaki Sano

**Affiliations:** 1 Community Care, Unnan City Hospital, 699-1221 96-1 Iida, Daito-cho, Unnan 699-1221, Shimane, Japan; yoshiyoshiryuryu.hpydys@gmail.com; 2 Department of Community Medicine Management, Faculty of Medicine, Shimane University, 89-1 Enya cho, Izumo 693-8501, Shimane, Japan; sanochi@med.shimane-u.ac.jp

**Keywords:** COVID-19, social norms, rural population, older people

## Abstract

Coronavirus disease 2019 (COVID-19) has affected people’s social lives by inhibiting their movement; this seriously impacts the lives of older people in particular. Rural older people may have been particularly affected because they live dispersedly and in isolation. This study explored rural older people’s perceptions of how COVID-19 has impacted their social lives. This qualitative study assessed participants who were 65 years and older and residing in rural Japanese communities. Five focus group discussions were conducted with 53 participants to explore their perceptions and challenges during COVID-19. Data were analyzed using thematic analysis, and four themes were developed: the beginning of suffering, social cognitive suppression, reflection on rural contexts, and critical approaches to the pandemic based on rural standards. The daily activities of rural older people were suppressed due to social norms and pandemic-related standard precautionary measures based on urban areas. Specific infection control standards for rural areas and the provision of direct information to individuals in the community to sustain social support are needed. To effectively maintain rural social support, as well as the trust and accountability of rural citizens, constant dialog among local governments and rural citizens is required.

## 1. Background

The coronavirus disease 2019 (COVID-19) pandemic has forced countries to implement policies to prevent the spread of infection by inhibiting citizens’ movements [1,2,3]. COVID-19 spread worldwide and resulted in many casualties, especially among older people [4]. Each country has adopted various approaches, such as implementing lockdown measures and declaring a state of emergency [1,2,3]. Despite constant efforts to control the spread of the virus, it continues to spread; this is a serious issue because COVID-19 has a high mortality rate among older people in particular [5]. Though the virus has mainly spread in urban areas, similar methods were used to control its spread in other areas [6]. Even though rural areas do not have many COVID-19 patients, they might be under the same administration as urban areas [7,8]. Furthermore, even though there is a gap in the status of the spread of COVID-19 between urban and rural areas [9,10], people in rural areas are being regulated by the same governments. Despite the methods to control the spread of the virus being mitigated and fitted to rural areas, such regulations have significantly impacted rural older people’s social lives; furthermore, these may persist even after the mitigation [11].

COVID-19 precautionary procedures can especially impinge on older people’s social lives and have a negative effect on their physical and mental well-being. Social isolation can affect mental health [12,13]. As many older individuals from rural backgrounds live alone or with their spouses, they depend on their distant families and community members for support [14,15]. During the pandemic, they might feel lonely and isolated from their society due to the implementation of lockdown regulations and social distancing [16,17,18]. Various studies have shown that there has been an increase in the number of older individuals with depression or anxiety disorders during this pandemic [19,20,21]. Social isolation also inhibits older people’s physical activities due to the lack of opportunities to go out and engage in community activities [22,23]. The lack of physical activity can affect their health and cause medical problems such as chronic disease exacerbation [24,25]. Controlling COVID-19 is critical; however, social interaction and physical activities should be encouraged within communities, depending upon the severity of their conditions regarding COVID-19 [26]. Moreover, in rural areas with fewer infections, there could be a relaxation in the restrictions and control measures to encourage social activities.

The end of this pandemic may not be predictable; therefore, the social lives of older people should be modified, and social activities should be encouraged among them by taking measures that adhere to global standards [27]. Furthermore, as this pandemic has induced tremendous fear among rural older people and placed a burden upon them, social norms might develop among the population regarding taking precautionary measures against COVID-19 [28]. Social norms refer to concepts and ideas that influence people’s actions in groups and in the current climate, the social norms related to COVID-19 might inhibit social activities in communities [28,29]. These norms can be formed based on specific contexts and indigenous backgrounds [29,30]. To encourage safe and practical physical activities among rural older people, social norms that inhibit their social activities should be identified. Therefore, our research questions were: How have rural older people experienced the COVID-19 pandemic? and What are the limitations and challenges regarding social activities among the rural population? In present conditions, various studies have shown the relationship between the pandemic and older people’s health [22,23,24,25], but few have explored the pandemic’s impact on rural older people’s social activities and related difficulties [31]. The present study inductively investigated rural older people’s perception of the COVID-19 pandemic, particularly related to changes in their social lives and their present challenges.

## 2. Methods

### 2.1. Setting

This study was performed in Unnan City, Shimane Prefecture, Japan. Unnan City is in the eastern part of Shimane and borders Hiroshima Prefecture from the south. Its total land area measures 553.1 km^2^ and accounts for 8.3% of Shimane, most of which is covered with forests. A survey conducted in 2017 revealed that the total population of Unnan City was 38,882 (18,720 men and 20,162 women), with 37.82% of the population being older than 65 years of age. There are 30 multifunctional autonomies in Unnan City, each of which has various functions for managing their respective social issues. In the traditional protocol, multiple community groups have specific functions (e.g., community organizing, healthcare, and continual education) [32]. Thus, each autonomous community organization comprises a director, sub-directors, and clerks who are active in three main categories: community organizing, healthcare, and social/environmental development. Community organizing refers to citizen empowerment, the effective utilization of local resources, and solving community problems through local actors’ efforts. The number of COVID-19 cases has been consistently lower than in urban areas such as Tokyo and Osaka. As of 31 December 2020, the total number of cases was 156 (0.022%), compared with 60,177 (0.65%) in Tokyo. All of them recovered, and there were no casualties.

### 2.2. Participants

The participants were 53 residents of Unnan City. Samples of voluntary participants were obtained from each autonomous community by sending invitation letters to people older than 65. The participants were organizers and participants of regular recreational activities offered in each community. Six communities (Kakeya, Tane, Matsukasa, Iruma/Anami, Hata, and Tai) were chosen based on them being a substantial distance away from the central parts of Unnan City. The study participants from each community center were briefed about the study, and they gave their informed consent for participation in the research. Before the participation, all participants were screened for fever or other COVID-19 symptoms. Participants also wore face masks, and a distance of more than two meters was maintained between the participants.

### 2.3. Data Collection

We conducted focus group interviews, each about 90 min long, in each community from 1 September to 31 December 2020. Focus groups were facilitated by the first author and audio was recorded, after which their contents were transcribed verbatim. The authors analyzed the transcriptions based on a thematic analysis. The interview guide included four main questions: What do you think about the difficulties faced in this pandemic? What do you think about the influence of this pandemic on your lives? How do you think you can modify the feeling of fear related to COVID-19? What do you think that we should do to overcome our difficulties? Five focus group discussions were conducted with older people in rural communities. Each group consisted of 8–12 of the 53 participants; 43.2% of the participants were male, and the average age was 78.2 years (standard deviation = ±5.6).

### 2.4. Data Analysis

Thematic analysis was used to identify rural older people’s perception of the conditions of the COVID-19 pandemic, the changes in their social lives, and present challenges regarding the limitations related to rural social activities [33]. The first and second authors carefully studied the interview transcriptions. Subsequently, the first author coded the content and developed codebooks based on repeated reading. The second author independently coded the contents. The two authors discussed the coding based on the codebook among themselves. In this process, the authors inducted, merged, deleted, and refined concepts and themes by comparing research materials and coding [33]. Discussions regarding the data and coding continued until mutual agreement was reached and no new concepts and themes were developed [33]. Finally, the themes and concepts were discussed and agreed upon by all authors. Furthermore, the result was translated from Japanese to English.

### 2.5. Ethical Considerations

Before providing written consent, participants were informed that the data would only be used for research purposes. They were also informed about the research aims, data disclosure procedures, and steps taken to protect personal information. This study was approved by the Unnan City Hospital Clinical Ethics Committee (approval number: 20200025).

## 3. Results

Four themes and ten concepts were developed as a result of the thematic analysis (Table 1).

### 3.1. The Beginning of Suffering

The suffering of rural older people began when the first cases of COVID-19 were detected in their communities, suddenly engendering fear among them. Following the first diagnoses, people began cloistering themselves, which caused severe disruptions to their daily lives. Furthermore, as the news coverage regarding COVID-19 increased, their fear grew drastically.

#### 3.1.1. Fear of Infection Based on Proximity

Having a COVID-19 case in their community changed the participants’ perceptions of the virus and made the infection seem more real. Rural older people might have initially felt fearful, but the emergence of COVID-19 cases around them seemed to intensify their fear. One of the participants stated:

“The first case of COVID-19 overwhelmed me, because I felt anxious but did not think that the infection would not have appeared in my community. That case changed the circumstances in the communities regarding COVID-19. A lot of older people must have feared the infection in real life.”

(Focus group E, participant 1)

COVID-19 cases affected daily life in rural communities, especially among older people. They were aware of the high mortality rate of COVID-19 among older people; thus, their activities were restricted because of a fear of infection. One of the participants stated:

“I could not go out of my home even when there was no infection in my community. I am old, so if I get infected, I may have serious symptoms. I felt a lot of fear and even now, I am reluctant to go out of my home.”

(Focus group B, participant 4)

#### 3.1.2. Disruption in Daily Life

Older people struggled to carry out their daily activities during the pandemic. Many could not drive, and public transportation was insufficient; therefore, they had to ask others to help them to travel to places. However, they were reluctant to ask others for help because of the anxiety related to getting infected and because they were conscious of what other people would think about the possibility of them being infected. One of the participants stated:

“I did not have my car and lost the license to drive because of aging. I had to ask friends to take me shopping, but I was reluctant to ask. I tended to think about how others thought about my condition, although I was not infected. Older people might have the same situation.”

(Focus group A, participant 7)

#### 3.1.3. Fear Triggered by Exposure to COVID-19-Related News

Participants were exposed to a lot of information related to the pandemic through mass media; the news mainly reported on urban areas where many patients were admitted in hospitals and hospital staff were exhausted. The rural older people noticed the vast differences between rural and urban regions regarding the spread of the infection. However, frequent exposure to news seemed to trigger their fear. One of the participants stated:

“Every day, there is tons of news regarding the coronavirus. Most news was from urban areas, not rural areas. However, the news was very sensational and induced fear in me. Although rural areas are safe with standard precaution, I tended to feel too much fear.”

(Focus group B, participant 3)

Older people were exposed to coronavirus-related news through various sources. They seemed to be frustrated by the repeated exposure to information. One of the participants stated:

“Not only from TV but also from the newspaper, radio, and calls from my relatives, the coronavirus news reached me. I could not escape from the news of coronavirus anywhere. Although there were no patients in the rural area, I had to fear inappropriately about COVID-19. I was fed up with the news.”

(Focus group D, participant 6)

### 3.2. Social Cognitive Suppression

Through continuous exposure to information regarding COVID-19, a new social norm was created even though there were few cases of infection. People in rural areas felt that they were supposed to cloister themselves in their homes, and they felt they risked social discrimination if they frequently left their homes and met others. The new social norm and the fear of social discrimination impinged on the lives of older people in rural areas.

#### 3.2.1. Created New Social Norm

The participants reported being reluctant to go out and preferred to stay at home. They tended to be anxious about other people’s perception of their behaviors and tried to follow other people’s actions, which led to them staying at home in quarantine for longer periods of time. One of the participants stated:

“I hoped to go out and meet others who were not at risk of infection. However, I didn’t. I felt that I was considered insane by others outside my home. Such feelings inhibited me from going out.”

(Focus group A, participant 5)

Rural social norms prevented participants from going out; this led to reduced social interactions. Due to repeated exposure to the news via mass media and the government’s information broadcasts, many rural people thought that going out would be dangerous. As rural societies are small and human interaction was limited, due to self-isolation, quarantine became a social norm. One of the participants stated:

“The present atmosphere is very heavy, especially for older people. Rural society might create an atmosphere in which people should not go out of their homes. It can be a new social standard, but it should be changed.”

(Focus group B, participant 9)

#### 3.2.2. Risk of Social Discrimination

As rural communities are small and citizens have close relationships with each other, rumors tend to spread faster than in urban settings. As the rural older people had lived in the community for a long time, they were aware of how fast rumors tend to spread. Fear of rumors inhibited rural older people’s lives because they feared that rumors could lead to social discrimination. One of the participants stated:

“Rumors are dangerous for rural people. If I were suspected of being infected with COVID-19, I could not live here. Usually, close relationships among citizens are good for our lives in a rural setting, but it can be dangerous in this pandemic. I know that I will not be infected by not going out from rural communities. My unusual behaviors can trigger rumors, which might impinge on our usual lives in the communities and cause others to discriminate against us.”

(Focus group A, participant 1)

Though intimate connections with others and social norms are crucial for the participants’ health and lives, the participants seemed to inhibit their activities during the pandemic.

### 3.3. Reflection on Rural Contexts

The daily difficulties they experienced drove the older people in rural areas to reflect on their situations. They had confidence in their appropriate understanding of precautionary measures and considered the social restrictions placed on them to be extreme and beyond what was necessary. When reflecting on the differences between rural and urban areas, they believed that their conditions should have been based on the actual rural conditions related to the pandemic.

#### 3.3.1. Appropriate Understanding of Precautionary Measures

The rural older people became used to infection control methods such as wearing a face-mask, handwashing, and meeting people from their communities and other communities only after confirming whether they had symptoms or were in contact with a COVID-19 patient. One of the participants stated:

“At first, I did not think that I could carry out infection control measures, masking, and so on. Now, we know how to deal with various aspects of coronavirus. I feel that I do have a tiny possibility of infection under the current scenario, and others in our communities may have confidence not to be infected.”

(Focus group B, participant 11)

Consistent efforts in taking precautions made rural older people confident about controlling the infection rate and made them feel safer. One of the participants stated:

“Getting used to the infection control may lead to my confidence to continue present procedures against infection. For nine months, many people were infected in the world and died, but my community had no patients. Our present effort is good, and I feel that we have a very low possibility of being infected.”

(Focus group A, participant 2)

#### 3.3.2. Feeling Extremely Restrained

The rural older people realized that they felt restrained due to the new social norms regarding the pandemic. They knew that controlling the spread of the infection was essential; however, they felt that their social conditions should be improved in the future. One of the participants stated:

“There are no patients with COVID-19. I do not understand why I have to cloister myself in my community. I understand that I should perform standard infection control, such as masking and washing hands. It should be continued for the future to prepare for unusual things. However, the present social suppression of older people should be revised.”

(Focus group D, participant 9)

Their reflections on their experiences during the pandemic changed their perception of managing the spread of COVID-19. The rural older people realized that they were able to do their part to control the infection by taking precautionary measures and that their feelings of restraints were not appropriate.

#### 3.3.3. The Difference between Urban and Rural Areas

Upon reflection, the participants were able to critically appraise the difference between urban and rural areas in terms of COVID-19. The rural older people’s fear of infection was instigated by the news spread by media. However, they realized that there was a vast difference between urban and rural areas; therefore, they were able to objectively think about the pandemic. One of the participants stated:

“There is an absolute difference between urban and rural areas regarding this infection. These days, I feel that there is a huge population in urban areas, as the news and movies show. This situation cannot prevent the spread of this infection. By seeing those around me, there are a few people, and there is natural social distancing.”

(Focus group B, participant 2)

Older people also realized the positive aspects of rural areas that can be beneficial to their health. One of the participants stated:

“I can go out to my farm and rice field because there is no person except for me. I can refresh my feelings and move my body. This cannot be the case in urban areas. We should respect present situations in rural areas.”

(Focus group C, participant 6)

### 3.4. Critical Approaches to the Pandemic Based on a Rural Standard

When the older people in rural areas reflected on their lives, they critically appraised their conditions and hoped for standards specific to rural areas. To accomplish the creation of the standards and the continuity of rural communities in this pandemic, they feel that dialogue within the communities is particularly important.

#### 3.4.1. Standards Specific for Rural Areas

Rural older people expressed hope for the development of standard precautionary measures specific to the rural context for carrying out their activities. They hope to be able to act freely in their communities, while keeping in mind the standard precautionary measures and the differences between rural and urban areas. One of the participants stated:

“I hope that the central and local governments develop the standard of cloistering respecting not only the number of the infections but also population density and usual lifestyles. In rural areas, people do not frequently meet others. Most people meet the same community people. In particular, local governments should respect our living conditions and make standards specific for rural areas.”

(Focus group E, participant 2)

#### 3.4.2. Importance of Dialog within the Community

During the pandemic, the rural older people realized the importance of daily conversations they had with others within their communities. Before the pandemic, these conversations usually took place at regular gatherings or recreational meetings organized by community centers and were considered routine activities in their lives. However, as they were unable to engage in conversation due to the precautionary measures, they began to realize its importance. One of the participants stated:

“I did not think that talking with others was so important in my life. I might have taken it for granted. However, after this pandemic, I lost most of the time to meet and talk with them. Reflecting on the talks, I realized the importance of meeting with others.”

(Focus group C, participant 10)

Furthermore, participants also realized the importance of being connected with others in rural communities. The pandemic hindered their naturally constructed social support and capital, which is maintained through constant dialog in rural communities. One of the participants stated:

“The social distancing in rural areas inappropriately destroyed our relationships leading to social support. I do not want to lose close relationships in my community, which have been constructed over a long time. I got old, so I could not keep up with various new technologies. I hope that I am able to regain opportunities to others and sustain the social support specific for rural areas.”

(Focus group A, participant 6)

## 4. Discussion

The results of this study showed that rural older people were initially overwhelmed with fear due to social isolation resulting from COVID-19 regulations. However, reflecting on the severity of the infection in rural areas mitigated their fear and led them to consider regaining opportunities in the future to meet others in their rural communities. Furthermore, rural older people felt restrained by the current standard precautionary measures, which were decided upon by the central government. They hoped to have discussions about the effectiveness of these standards in a rural context.

For the participants, feelings of fear at the beginning of the pandemic seemed unmanageable because of the unknown and quick-spreading virus. This fear was exacerbated by unfamiliar precautionary measures and the news conveyed by mass media. COVID-19 rapidly spread worldwide, and information regarding its treatment and vaccinations has been constantly changing [34,35,36]. In the past, epidemics—such as severe acute respiratory syndrome (SARS) and Middle East respiratory syndrome (MERS) [37,38]—instilled fear in the participants because there was a lack of information regarding the transmission and severity of infection [39]. COVID-19 differed from these previous outbreaks, as it was a pandemic that rapidly spread throughout the world. The SARS virus and MERS virus spread broadly in Asian countries but not in America and Europe [40]. As COVID-19 spread, each country had to independently manage the virus [41], and different countries adopted different approaches to address it [3,42]. Mass media provided a lot of information on the virus from different sources [43,44,45]. This study’s participants stated that they felt confused due to the different (sometimes contradictory) information from different government, health, and news sources. Large amounts of information regarding the virus through mass media overwhelmed rural older people [46,47]. Considering these experiences of older rural residents, one way to address this is for the World Health Organization to control the pandemic information being circulated. Moreover, health-related organizations in each country should direct citizens to act according to standards specific to the severity of COVID-19 in that particular area.

New social norms that were formed because the fear of COVID-19 disrupted rural older people’s social lives; this can be explained through the social cognitive theory. Bandura’s social cognitive theory states that human behaviors are governed by not only their perceptions of their own behaviors but also by others’ interpretation of their behavior and the social norms in their communities [48]. This process occurs through reciprocal determinism, which means that an individual’s behavior can influence and be influenced by others in their community [48]. The participants stated that they were highly conscious of other people’s perceptions of their behavior when they were going out of their houses during the pandemic. The social norm formed in the community during the pandemic was that going out of one’s home is dangerous and should be avoided. This inhibited rural older people’s behaviors [49]. It is possible that other people’s perceptions are given more importance in rural areas compared with urban areas because reciprocal determinism is stronger when the relationships are close [50]. While individuals in urban areas might be losing close human relationships because of the lack of interaction with others, residents of rural areas have strong human relationships, as shown in this study [50]. In terms of controlling the pandemic, reciprocal determinism can be useful because an individual’s behavior can influence and be influenced by others in their community [29]. However, suppressing the physical activity of rural older people also led to a deterioration in their mental health. This study’s participants stated that they struggled to continue with their daily lives because of a lack of accessibility and being unable to ask for help from younger people. The formation of new social norms as a result of this pandemic may be inevitable; however, we must consider the strong effect of social norms in rural areas and the mitigation of restrictions in rural contexts to form and maintain healthy social norms in rural settings.

Reflecting on the status of the pandemic in the rural context enabled the participants to understand it better, and they were able to contemplate ways to effectively sustain social support. The participants were confident about the precautionary measures against COVID-19 being taken in their community. A previous article regarding the Ebola outbreak suggests that reflecting on one’s situation considering one’s specific context is vital for effective infection control [51]. The infection spread was more prevalent in urban and congested areas, and rural older people realized that rural areas are not congested; therefore, there is a lower risk of infection. To effectively control the spread of COVID-19, local governments and citizens should reflect on the status of COVID-19 in their specific areas [52]. Furthermore, as this study’s results suggest, continued discussion among local governments and rural citizens is required to effectively sustain rural social support based on community relationships. Previous research has suggested that adequate infection control provision requires the involvement of local people and is based on trust and accountability [51,53]. To ensure the participation of rural citizens, local governments should involve them in discussions regarding the pandemic and seek their help to assess the status of the pandemic in rural contexts. The role of rural citizens in the sustainability of rural social support should be identified. This will improve the trust and accountability of older rural citizens.

Despite its clear contributions, this study had certain limitations. A limitation of this research is its transferability. We focused on rural older people in a Japanese context. Even though Japan has relatively fewer patients with COVID-19, rural areas in other countries might be dealing with similar conditions, with a lack of medical resources and fewer people in less congested areas. The findings of this study can be generalized to other rural contexts, not only to help control the spread of the virus but also to examine the nature of strong and effective social norms. Another limitation is its dependability. This study was performed during the third wave of the COVID-19 pandemic in Japan, and participants had experienced various stages of the pandemic. The perception of citizens could have changed with time. In this study, the authors attempted to examine the changes in their perception regarding COVID-19. The changing perceptions of rural citizens can also be generalized to other rural contexts. Furthermore, because this study used focus group discussions and did not quantitatively assess rural older people’s health conditions, future research should quantitatively explore changes in their health conditions with respect to quality of life and by using one-on-one interviews to determine personal factors regarding the fear of COVID-19 in rural settings.

## 5. Conclusions

This study shows that while overcoming their fear of the COVID-19, the daily activities of rural older people were suppressed as a result of the application of social norms and standards that are based on urban areas. There is a need to establish appropriate precautionary measures for rural settings and provide information to sustain social interactions, particularly among older people. To effectively sustain rural social support and the trust and accountability of rural citizens, constant dialog among local governments and rural citizens is required.

## Figures and Tables

**Table 1:** Themes and concepts regarding rural older people’s perception of the conditions during the COVID-19 pandemic.

Theme	Concepts
The beginning of suffering	Fear of infection based on proximity
	Disruption in daily lives
	Fear triggered by exposure to COVID-19-related news
Social cognitive suppression	Created new social norm
	Risk of social discrimination
Reflection on rural contexts	Appropriate understanding of the precautionary measures
	The difference between urban and rural areas
	Feeling extremely restrained
Critical approaches to the pandemic based on rural standard	Standards specific for rural areas
	Importance of dialog the within the community

COVID-19: coronavirus disease 2019.

## Data Availability

The datasets used and/or analyzed during the current study are available from the corresponding author upon reasonable request.
